# Pathogenic Mechanisms in Cervical Cancer: Energy Metabolism, Hypoxia and Therapy

**DOI:** 10.3390/life16030450

**Published:** 2026-03-10

**Authors:** Valentina Giorgio, Valentina Del Dotto, Martina Grandi, Silvia Grillini, Giancarlo Solaini, Alessandra Baracca

**Affiliations:** Department of Biomedical and Neuromotor Sciences, University of Bologna, I-40126 Bologna, Italy

**Keywords:** cervical cancer, mitochondria, metabolic reprogramming, hypoxia, non-coding RNAs, therapy

## Abstract

Cervical cancer has a high incidence and mortality, and is one of the leading causes of cancer-related deaths among women worldwide. The infection with high-risk subtypes of the human papillomavirus (HPV) represents a crucial factor in the development of precancerous lesions. HPV oncoproteins target multiple host factors to promote uncontrolled cellular proliferation, genomic instability, profound metabolic reprogramming, resistance to apoptosis and immune evasion. Thus, cervical carcinogenesis involves metabolic reprogramming in patient cells, such as enhanced aerobic glycolysis, and altered glutamine, lipid and mitochondrial metabolism, which collectively support the bioenergetic and biosynthetic demands of cancer cells. Cancer cells also activate several mechanisms to adapt and survive under hypoxic/anoxic conditions. The mechanisms underlying cervical carcinogenesis often involve non-coding RNAs. This review aims at summarizing the mechanisms and factors involved in the development and progression of cervical cancer following HPV infection, and offers an overview of the available therapies that have been developed for this disease.

## 1. Introduction

Gynecological cancers, which include ovarian, endometrial and cervical cancer, are the highly occurring cancers in females and, globally, account for more than one-third of the newly diagnosed cancers [1]. In particular, cervical cancer represents a major public health problem, ranking as the fourth most common cancer among women after breast, colorectal and lung cancers [2]. Cervical cancer has a high incidence and mortality, and is one of the leading causes of cancer-related deaths among women worldwide [1,3]. Indeed, although its incidence has decreased in the last three decades thanks to vaccination, screening and treatment of precancerous lesions, cervical cancer accounted for around 51% of deaths due to gynecological malignancy and remains the second leading cause of cancer death in women aged 20 to 39 years [1,4,5].

Persistent infection of human papillomavirus (HPV) is detected in more than 90% of all cervical cancers and is recognized as the primary cause [2,6,7]. Among the over 200 identified HPV genotypes, 40 infect the genital tract and 17 subtypes have been classified as high-risk based on their potential to induce invasive cervical cancer [3]. In particular, HPV16 accounts for the highest global cervical cancer cases (around 60%), whereas HPV18 is responsible for approximately 15% of cervical cancer cases. The most common histological types of cervical cancer are squamous cell carcinoma (75–80%), in which HPV16 is the predominant genotype, and adenocarcinoma of the cervix (15–20%), in which HPV18 is more frequently detected [2,3].

The infection with high-risk subtypes of HPV is a crucial factor in the development of precancerous lesions, but it is not sufficient by itself. Indeed, initiation and progression may be modulated by additional host- and environmental-related cofactors, including genetic mutations, chronic inflammation, vaginal microbiome, conditions associated with immunosuppression and dietary or lifestyle factors [1,8,9].

Persistent HPV infection allows the integration of viral DNA into the host genome, a key event in cervical carcinogenesis, as it enables the continuous expression of viral oncoproteins [10,11,12]. HPV is a circular, double-stranded DNA virus and its genome is divided into three regions: early (E), late (L), and long control region (LCR). The LCR contains regulatory elements controlling viral transcription and replication, the L region encodes structural proteins, and the E region encodes E1–E7 proteins, which are essential for viral replication, transcription, and cellular transformation [13]. Among the E proteins, E6, E7 and, to a lesser extent, E5 play pivotal roles in malignant transformation. Indeed, the coordinated activities of these HPV oncoproteins target multiple host factors to promote uncontrolled cellular proliferation, genomic instability, profound metabolic reprogramming, resistance to apoptosis and immune evasion [12,14]. These mechanisms not only enable the development of cervical cancer, but also accelerate metastasis and resistance to therapy [12,15].

## 2. Energy Metabolism in Cervical Cancers

Cervical carcinogenesis involves metabolic reprogramming in patient cells. Differential analysis of metabolites from cervical cancer, cervical intraepithelial neoplasia and control tissues showed changes, indicating a rewiring of oxidative glycolysis (the Warburg effect), pentose phosphate pathway, amino acid and lipid metabolism to meet the high energy requirements of virus production and promoting cancer cell replication [16,17].

HPV oncoproteins, involved in such metabolic alterations, further affect the cellular redox balance by increasing the levels of reactive oxygen species (ROS) [18,19], resulting in host oxidative stress and DNA damage, thereby facilitating viral DNA integration and causing neoplastic transformation [18]. The downstream targets of HPV oncoproteins are summarized in Table 1.

### 2.1. Glucose Energy Metabolism

**Figure 1:**
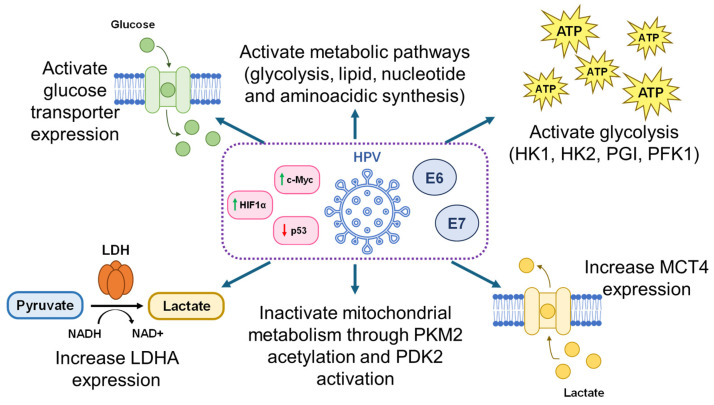
**Overall** **regulation of glucose metabolism mediated by E6 and E7 HPV oncoproteins and by p53, c-Myc, and HIF1α signaling**—E6 and E7 HPV oncoprotein expression, through p53 downregulation and c-Myc and HIF1α upregulation, drives metabolic reprogramming toward aerobic glycolysis by enhancing glucose uptake, increasing the expression of key glycolytic enzymes and Lactate Dehydrogenase A (LDHA), suppressing mitochondrial oxidative metabolism via Pyruvate Kinase Isozyme M2 (PKM2) acetylation and Pyruvate Dehydrogenase Kinase 2 (PDK2)-mediated inhibition of pyruvate dehydrogenase, and upregulating the Monocarboxylate Transporter 4 (MCT4) to facilitate lactate release. Green and red arrows indicate upregulation or downregulation, respectively.

Cancer metabolism is known to affect glucose utilization and mitochondrial function. It generates a complexity of metabolic phenotypes depending on each specific cancer; however, it is known that cervical cancer patients adhere to the traditional Warburg effect [20]. HPV downstream targets, such as p53 and c-Myc, have been shown to control metabolic regulators that contribute to the Warburg phenotype, promoting glycolysis under oxidative conditions (Figure 1) [21]. While p53 is downregulated, c-Myc has been reported to be activated in cervical carcinomas [21].

P53, a key player in metabolic regulation, is one of the crucial targets of the HPV protein E6 [22]. In normal conditions, p53 limits glycolysis in cells [23] by targeting the glucose transporters GLUT1 and GLUT4, reducing glucose uptake through the inhibition of the insulin receptor (INSR) and through the suppression of NF-kB, through which p53 can indirectly downregulate the expression of GLUT3 [24]. Finally, p53 limits glucose transport by directly activating the transcription of Ras-related glycolysis inhibitor and calcium channel regulator (RRAD), which prevents GLUT1 from translocating to the plasma membrane [25]. Thus, p53 degradation by E6 seems to increase glucose transporters, suggesting an increase in glucose uptake.

Glycolytic enzymes, such as hexokinases 1 and 2 (HK1, HK2), and glucose phosphate isomerase, which are normally inhibited by p53 [26], are suggested to be activated by the E6-mediated downregulation of p53 [15]. Moreover, HK2 is upregulated by *MYC* transcript stabilization upon HPV infection [27]. The HPV E7 oncoprotein promotes the acetylation of the pyruvate kinase M2 isoform (PKM2), the enzyme which catalyzes the conversion of phosphoenolpyruvate to pyruvate, inducing a protein conformation with less affinity for phosphoenolpyruvate, which results in a substrate accumulation [28].

HPV oncoproteins also promote the PI3K/AKT/mTOR pathway, a major regulator of cell growth and metabolism [29], through the activation of lipid synthesis, glucose absorption, and glycolysis, while inhibiting glycogen production [30]. Moreover, HPV-induced hyperactivation of AKT upregulates the expression of the glycolytic enzyme phosphofructo-kinase 1 (PFK1) and enhances the expression and membrane translocation of glucose transporters [31].

The aforementioned HPV oncoproteins promote glutamine metabolism in order to favor the biosynthesis of nucleotides and amino acids [32]. They also stimulate the pentose phosphate pathway, which is crucial for the biosynthesis of nucleotides and lipids [33].

HPV-positive HeLa and SiHa cells also showed increased uptake of glucose and glycolytic intermediates, such as glucose-6-phosphate, fructose-6-phosphate, and lactate, as well as increased levels of ribose, a pentose phosphate pathway intermediate, and ribitol, a pentose formed by the reduction of ribose [34].

During aerobic glycolysis, cancer cells preferentially convert glucose to lactate [35], the accumulation of which has been shown to activate c-Myc [36] and stabilize transcription factors such as HIF, affecting several metabolic changes that are described below.

Pyruvate is converted to lactate by the enzyme lactate dehydrogenase (LDH) in the cytosol [37,38]. This reaction allows cancer cells to continue glycolysis due to the regeneration of NAD^+^, which is necessary for the glycolytic reaction catalyzed by glycerleide-3 phosphate dehydrogenase. Numerous HPV targets, such as p53, HIF, and c-Myc, stimulate the production of the LDHA isoform of LDH enzymes [15]. HPV E6 oncoprotein also promotes the increase in lactate by upregulating LDHA levels through the activation of the mTOR signaling pathway [39]. Moreover, LDHA has also been reported to be a c-Myc-responsive gene [40].

Lactate efflux into the extracellular matrix is mediated by monocarboxylate transporter (MCT) isoforms 1–4 of the MCT family [41]. The expression of MCT4, which mediates lactate release, was found to be increased along progression to malignancy in cervical cancer patients [42,43]. MCT1, which is downregulated by p53 [44], was suggested to be increased upon p53 degradation induced by E6 HPV oncoprotein [15]. However, low levels of MCT1 were detected in patient samples [42].

An increase in lactate and its accumulation even in the presence of oxygen causes an acidic internal environment and extracellular tumor micro-environment, causing interactions with tumor-associated fibroblasts (TAFs) and promoting angiogenesis, metastasis, and immune response evasion [38,45]. An acidic environment stimulates the release of the angiogenic factor VEGF, epithelial-to-mesenchymal transition (EMT) factors such as vimentin, β-catenin, which is also found in HeLa cells during migration [46], or the transforming growth factor-beta (TGF-β) [38].

The metabolic shift towards glycolysis has been shown to correlate with worse prognosis in patients with cervical cancer [47,48].

### 2.2. Mitochondrial Energy Metabolism

**Figure 2:**
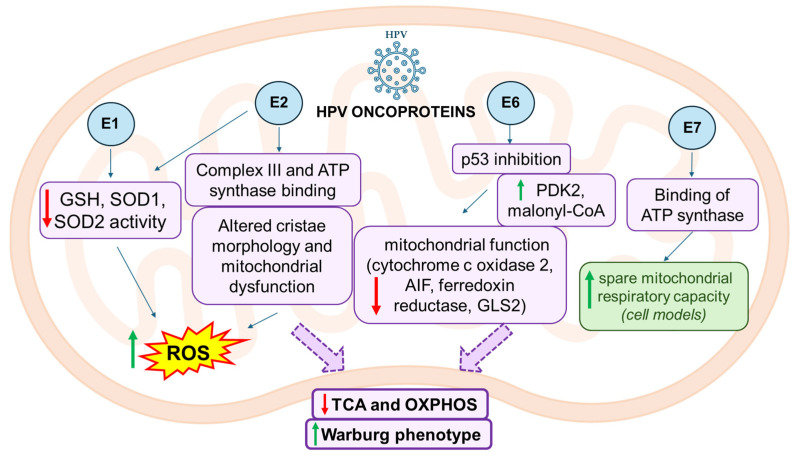
**Schematic** **representation of mitochondrial metabolism in cervical cancer and its regulation by HPV oncoproteins**—HPV oncoproteins E1, E2, E6, and E7 activate host mitochondrial signaling pathways that increase intracellular ROS levels, impair mitochondrial function, promote lipogenesis and regulate glutamine metabolism; collectively, these alterations downregulate TCA cycle (TCA) and oxidative phosphorylation (OXPHOS), thus promoting the Warburg phenotype. Green and red arrows indicate upregulation or downregulation, respectively.

Mitochondrial metabolic pathways, including the TCA cycle, fatty acid β-oxidation, and oxidative phosphorylation, are crucial for providing ATP and supporting cancer cell energy requirements [49,50]. In cervical cancer, several mitochondrial pathways appeared altered (Figure 2).

The E2 oncoprotein was shown to interact with the inner mitochondrial membrane, thus altering *cristae* morphology, which in turn affects cellular respiration and enhances ROS production [19].

P53 plays a crucial role in increasing the TCA cycle and oxidative phosphorylation. In normal conditions, p53 favors the expression of proteins, such as the cytochrome c oxidase 2 [51], the apoptosis-inducing factor (AIF) [52] and ferredoxin reductase, which are involved in the mitochondrial integrity. Therefore, p53 degradation, induced by the HPV E6 protein, inhibits these pathways at different points [18].

P53 suppresses the expression of pyruvate dehydrogenase kinase 2 (PDK2), a kinase that covalently deactivates the pyruvate dehydrogenase (PDH) [53]. The PDK2 deactivation induces the production of acetyl-CoA through the active catalytic activity of PDH, while its activation, under E6 control, inhibits PDH, disfavoring the TCA cycle and oxidative phosphorylation [18]. This activation causes pyruvate accumulation in the cytosol, resulting in lactate production [54]. A decrease in TCA cycle metabolites was confirmed in HPV-infected HeLa and SiHa cell models when compared with the HPV-negative C33A cellular model through a compelling metabolomic analysis [34]. Consistent with a decreased mitochondrial metabolism, these authors described lower levels of pyruvate and citrate, but high levels of NAD^+^, NADH and methylated 1-methylnicotinamide [34], suggesting a shift towards glycolysis and lactate production that may help host cells to maintain a high growth rate. On the contrary, however, other studies have suggested that increased levels of the enzyme responsible for methylation of nicotinamide to 1-methylnicotinamide (nicotinamide N-methyltransferase) result in the induction of mitochondrial activity and ATP synthesis and may play a key role in the sustained growth of SiHa [55].

Glutamine metabolism is also targeted by the HPV E6 oncoprotein. P53 promotes the expression of glutaminase 2 (GLS2), an enzyme that converts glutamine to glutamate, which, in turn, is converted into α-ketoglutarate supplying the TCA cycle [56]. This has been suggested as another mechanism that the HPV infection might disfavor, inducing the Warburg phenotype in infected cervical cells [18].

HPV oncoproteins also modulate lipid metabolism by activating enzymes involved in de novo lipogenesis, thus supporting lipid accumulation and providing energy for tumor growth [17,57]. The transcription of malonyl-CoA decarboxylase (MCD), which converts malonyl-CoA to acetyl-CoA, is promoted by p53 [58]. A reduction in p53 levels, due to HPV E6 oncoprotein, leads to the downregulation of MCD and consequent accumulation of malonyl-CoA, which is an allosteric inhibitor of carnitine palmitoyl-transferase 1 (CPT1) [59], the rate-limiting enzyme in fatty acid β-oxidation. Thus, E6 action on p53 also limits β-oxidation and acetyl-CoA production, which, in turn, limits the TCA cycle in mitochondria.

Insulin-like growth factor 2 mRNA-binding protein 3 (IMP3) regulates mitochondrial function and lipid metabolic programs in cervical cancer. IMP3 knockdown in HeLa cells reduced the mitochondrial oxygen consumption rate and altered the cellular energy status, as evidenced by changes in ATP/ADP and NADP^+^/NADPH ratios [60]. Transcriptomic profiles from normal cervical tissues, primary tumor and paracarcinoma donor samples, followed by protein–protein interaction network construction and metabolite analysis, associated IMP3 with coordinated changes in respiration and lipid metabolism in cervical cancer [60].

Martínez-Ramírez and coauthors suggested that E2 protein could regulate ROS release and mitochondrial activity through its interaction with complex III and ATP synthase [18], which also controls *cristae* mitochondria in mammals [61,62]. Moreover, it has been suggested that the co-expression of E2 and E1 from the HPV18 subtype increases ROS levels and DNA damage by decreasing GSH levels and the activity of both SOD1 and SOD2 [63], thus causing important events contributing to mitochondrial dysfunction [64] inducing carcinogenesis or promoting viral replication. The direct interaction of the HPV oncoprotein E7 with the β subunit of the mitochondrial ATP synthase upon infection of HPV8, HPV11 and HPV16 was shown [65]. This study also indicated a co-localization in mitochondria of the two interacting proteins in FLAG-tagged E7-transfected C33A cells. The β subunit–E7 interaction was followed by an increase in spare mitochondrial respiratory capacity in HPV8, and an even more evident increase in HPV16, E7-expressing cells, while a decrease in glycolysis was observed [65]. The β subunit was also shown as a key site for calcium binding in a HPV18-positive HeLa model which modulates apoptotic death [66].

Recent studies performed in HPV-positive HeLa cells showed that cervical cancer cells display a higher IF_1_/ATP synthase ratio in comparison with other carcinomas [67]. IF_1_, the endogenous inhibitor of ATP synthase, is upregulated in different cancers, where it can act with several oncogenic roles, including ATP hydrolysis inhibition under hypoxia/anoxia [68,69]. Beyond its main inhibitory function in hypoxia, described below, IF_1_ has been associated to a number of other functions reported and analyzed in recent reviews from our laboratory [68,69]. Indeed, in HeLa cell models it has been shown that IF_1_ plays a role in cell survival by inhibiting apoptosis through two different molecular mechanisms: contributing to the preservation of the *cristae* structure [70,71] and interacting with the OSCP subunit of ATP synthase, thus resulting in a desensitization to the permeability transition pore opening [72,73,74,75]. Furthermore, HeLa cells showed the highest ROS levels compared to other cancer cells [50], although they were characterized by the highest IF_1_/ATP synthase ratio, and IF_1_ was shown as a factor limiting ROS levels in other models [76]. Other studies showed that the inhibition of the first rate-limiting enzyme of glycolysis can alter the metabolic mode of HPV-positive cervical cancer, increasing mitochondrial function and causing a significant increase in the sensitivity of cervical cancer cells to radiation [77].

## 3. Adaptation of Cervical Cancers to Hypoxia

Cancer cell population of solid tumors is characterized by a heterogeneous energy metabolism due to various reasons, including the different distance from blood vessels, thus affecting the O_2_ availability [78,79]. Therefore, cells can be exposed to a condition of normoxia, oxygen tension (pO2) above 4%, a hypoxic condition, when the pO2 is below 4%, or severe hypoxia, at a pO2 below 1% [80,81]. Typically, the metabolic phenotype of cancer cells presents an enhanced glycolysis compared to normal cells even in the presence of physiological oxygen availability (Warburg effect), as described in the previous chapter. Hypoxia is a hallmark of the tumor micro-environment present in the vast majority of solid tumors [82]—as a consequence of an imbalance between increased oxygen consumption and inadequate oxygen supply—due to rapid cellular proliferation that exceeds the supply capacity of the vascular system because of an insufficient angiogenesis [83]. The latter is a condition of particular interest in studies of cervical cancer, which is characterized by a median pO2 of about 1% [84,85]. Indeed, hypoxia represents a prognostic factor associated with poor clinical outcomes and several hypoxia-related genes have been identified as biomarkers for predicting overall survival in patients with cervical cancer [86,87,88,89]. Hypoxia can lead to cancer resistance to therapies like radiation and chemotherapy [90,91], and may therefore have large therapeutic consequences.

When hypoxia occurs in cells, oxidative phosphorylation decreases the synthesis of ATP due to the decreased flow of electrons through the respiratory chain; when the oxygen concentration falls below a certain threshold, severe hypoxia or anoxia occurs. In these two conditions, the electrochemical potential (Δµ_H+_) of the inner mitochondrial membrane collapses, pushing the ATP synthase to work in reverse, thus hydrolyzing the ATP. The ATP molecules are supplied by the increased glycolytic flux, which is driven by the decreased energy charge. This allows cells to maintain a level of Δµ_H+_ that avoids mitophagy and/or death. Indeed, under this condition, normal cells can survive thanks to an endogenous inhibitor of ATP synthase, IF_1_, which limits the hydrolytic action of the enzyme, to balance the hydrolysis of ATP and the level of Δµ_H+_ [68]. Many cancer cells contain higher levels of IF_1_ than normal cells, which can completely inhibit the hydrolytic activity of the enzyme promoting cell survival, even in the presence of a collapsed Δµ_H+_ that energetically mimics the absence of significant oxygen supply. Moreover, IF_1_ expression promotes cell proliferation upon reoxygenation [92,93].

In cervical cancer cells, hypoxic conditions have been associated to several changes, including HIF1 activation, which, in turn, induces decreased apoptosis and autophagy, EMT, and overexpression of oncogenic non-coding RNAs, leading to increased cancer aggressiveness or resistance to therapy [91]. Indeed, in the presence of low oxygen levels, cells reprogram their metabolism by inducing the hypoxia-inducible factor HIF1, which is a heterodimeric transcription factor consisting of α and β subunits, first described by Semenza and Wang [94]. In the presence of low oxygen levels, HIF1α binds to HIF1β, forming the dimer HIF1 through a complex mechanism in which oxygen tension is critical [95]. The activated HIF1 interacts with the hypoxia-responsive elements on DNA, activating the transcription of several hundred genes, allowing cells to adapt to the hypoxic environment by metabolism reprogramming consisting *inter alia* in enhancing glycolysis and inhibiting oxidative phosphorylation, by modulating the expression of GLUT1, HK1, HK2, LDHA and PDH, respectively [96]. In addition, HIF1 promotes the expression of VEGF, Vascular Endothelial Growth Factor which induces angiogenesis, reversing the cell hypoxic stress [97].

Hypoxia is particularly relevant in cervical cancer, as the oncoproteins E6 and E7 act to modulate this pathway, with HIF1 as a key target. Indeed, E6 has been shown to directly interact with HIF1α, enhancing its stability by preventing proteasomal degradation [98]. E6 also promotes HIF1 activation indirectly by counteracting the inhibitory function of p53 [99] and by inducing mTORC1 signaling, which ultimately results in HIF1α accumulation [100,101]. Moreover, also the oncoprotein E7 can bind and upregulate HIF1 [102] and can increase HIF1 transcriptional activity by interacting with histone deacetylases (HDACs), thus reducing their inhibitory effects [103]. In addition, E7 upregulates the ribonucleotide reductase M2 subunit (RRM2) that, in turn, promotes HIF1α and VEGF expression through ERK1/2 signaling pathway activation, thereby contributing to angiogenesis in cervical cancer cells [104]. Therefore, this aspect plays a central role among therapeutic approaches for the treatment of cervical carcinoma.

Besides the above-described molecular mechanisms adopted by tumor cells, other interesting mechanisms have been reported to occur in hypoxic cervical cancer cells. A very recent investigation on both HeLa and SiHa cell lines found that hypoxia promoted the malignant phenotypes of the cells by enhancing glycolysis via the upregulation of the Octamer-binding Transcriptional factor 4 (OCT4). Its overexpression enhanced the transcription of Calcium Release-Activated Calcium Modulator 3, which, in turn, activated the calcium signaling pathway ultimately promoting the malignant progression of cervical cancer [105].

Another mechanism ascribed to hypoxia is the entry into the mitochondria of STAT5A, the signal transducer and activator. In the organelle, STAT5A has been shown to interact with and inhibit the pyruvate dehydrogenase complex, decreasing both glycolysis and the TCA cycle, thereby reshaping both cellular energy production pathways, glycolysis and oxidative phosphorylation [106]. The activity of pyruvate dehydrogenase was increased in STAT5A-KO HeLa cells compared with parental cells, and the reintroduction of STAT5A-WT into STAT5A-KO HeLa cells restored enzyme activity to levels similar to those in parental HeLa cells. This was described to strengthen the Warburg effect in cancer cells and promote cell growth in vitro under hypoxia and in vivo tumor growth. These findings indicate the distinct pro-oncogenic roles of STAT5A in energy metabolism, which is different from its classical function as a transcription factor [106].

Finally, it is worth mentioning that, under hypoxic conditions, overexpression of the pro-oncogenic protein CDK regulatory subunit 2 (CKS2) is suppressed, contributing to the metabolic shift towards glycolysis and the Warburg effect. This is quite important since the pro-oncogenic CKS2 is overexpressed and associated with aggressiveness of many human malignancies, including cervical cancers [107], and seems to have some function at the nuclear level [108]. Radulovic and colleagues [109] proposed that CKS2 may also have a mitochondrial function in complex with the cyclin-dependent kinase (CDK1) and the mitochondrial single-stranded DNA-binding protein SSBP1, which is a protein required for single-stranded DNA stabilization during mitochondrial DNA (mtDNA) replication. More recently, Jonsson M et al. proposed a mechanism for the mitochondrial function of the oncoprotein CKS2 by in situ proximity ligation assays [110]. CKS2 was suggested to form a complex with the positively correlated Myc target, mitochondrial single-stranded DNA binding protein SSBP1, in the mitochondria of cervix tumor samples and in the HeLa and SiHa cervical cancer cell lines. This indicates a role in mtDNA replication and establishes a link between the regulation of cell division by nuclear pathways and oxidative phosphorylation in the mitochondrion involving CKS2 and promoting chemo-radioresistance of cervical cancer, which hypoxic cells escape.

## 4. Key Non-Coding RNA Sequences Promote Cervical Cancer Progression

Non-coding RNAs (ncRNAs), including long non-coding RNAs (lncRNAs), circular RNAs (circRNAs) and microRNAs (miRNAs), may act with different functions in transcriptional, translational and post-translational regulation. Changes in the expression of ncRNAs in cervical cancers have been closely associated with disease initiation and progression, highlighting their role as key regulators of cervical tumorigenesis and as potential diagnostic markers. Although a variety of ncRNAs have been revealed in patients as promising biomarkers [111], the evidence for circRNAs is restricted to cell- and tissue-based studies [112]. Due to their relevance to the clinics, the following paragraphs are focused on the lncRNAs and miRNAs that are associated with pathogenic mechanisms occurring in cervical cancer patients.

### 4.1. LncRNAs in Cervical Cancers

Several studies identified lncRNAs that are modified in their expression levels by HPV infection and activate pathogenic mechanisms promoting cervical cancer. Liu et al. have reported that, following HPV infection, 194 lncRNAs are differentially regulated primarily by the HPV oncoprotein E7 and, to a lesser extent, by E6 [113]. Some of these lncRNAs are associated with metastasis, disease severity and poor prognosis [114,115]. The majority of lncRNAs are found to be overexpressed in the body fluids as well as in biopsies of cervical cancer patients [112]. The main mechanisms that are modulated by their altered levels are associated with the increase in cell proliferation, angiogenesis, control of apoptosis and migration through different mechanisms, including the upregulation of epithelial–mesenchymal transition markers, such as the Wnt/β-catenin pathway [112,116]. A few lncRNAs are known to act as tumor suppressors, and they were found to be downregulated in cervical cancer [112,117].

#### LncRNAs Modulate Glycolytic and Mitochondrial Metabolism in Cervical Cancers

Different lncRNAs are described as important mediators of the metabolic changes occurring upon HPV infection in cervical cancer patients (Table 2).

ANRIL—Relevant metabolic rewiring-related lncRNAs include Antisense Non-coding RNA in the INK4 locus (ANRIL). lncRNA ANRIL upregulation is associated with metastasis, cancer severity and poor prognosis, and its high expression can be evaluated as a prognostic indicator [114,115]. Its high levels are associated with the activation of the PI3K/AKT pathway [115], thus promoting the glycolytic pathway and glucose uptake.

CRNDE—The lncRNA Colorectal Neoplasia Differentially Expressed (CRNDE) is significantly upregulated in cervical cancer cells and tissues, where it seems to act as an oncogenic promoter, representing a promising therapeutic target [118]. Besides its effects in promoting cell proliferation, migration and invasion [119,120], its oncogenic activity can be mediated through the suppression of P53 Upregulated Modulator of Apoptosis (PUMA) signaling pathway [121], thus resulting in increased glycolysis and Warburg phenotype.

H19—The lncRNA H19 altered expression upregulates Sirtuin-1 (SIRT1), which, in turn, controls glucose and mitochondrial metabolism, promoting cervical cancer progression and inhibiting apoptosis [122,123]. Additionally, Zhao et al. (2022) showed that serum H19 levels are high in cancer patients and decreased after surgery, suggesting their potential as non-invasive biomarkers for diagnosis [157].

HOTAIR—HOX Antisense Intergenic RNA (HOTAIR) acts as a miRNA sponge, namely, for miR-29b, miR-143–3p, miR-206 and miR-331–3p. This lncRNA showed significant positive correlation with E7 expression in HPV16-positive cells, suggesting an interplay between E7 and HOTAIR which promotes cervical carcinogenesis [158]. Via the miR-29b/PTEN/PI3K axis, HOTAIR promotes EMT in cervical cancer [129]. Through miR-29b, HOTAIR can downregulate Phosphatase and Tensin Homolog (PTEN) protein levels, which leads to the sustained activation of the PI3K/AKT pathway [159,160], promoting glucose and lipid metabolism in cervical cancer. The HOTAIR-induced upregulation of genes associated with EMT, including VEGF and Matrix Metalloproteinase-9 (MMP-9), enhances the migratory and invasive properties [124,130]. Importantly, HOTAIR controls apoptosis and mitochondrial function in cervical cancer by binding to miR-143–3p, which indirectly modulates Bcl-2 expression [125]. HOTAIR deficiency was associated with an impairment of the mitochondrial respiratory chain and *cristae* loss, leading to depolarization of mitochondrial membrane potential and swelling [127,128].

LincRNA-p21—This is a previously identified p53-inducible lncRNA which was revealed to interact with HIF1α. LincRNA-p21 has been associated with different mechanisms of cancer biology, such as angiogenesis, mitochondria metabolism, cell survival, and tumor invasion [161,162]. LincRNA-p21, as a hypoxia-responsive lncRNA, plays a key role in hypoxia-induced glycolysis in various types of cancer, thus promoting tumor growth under hypoxia [132].

MALAT1—Metastasis-Associated Lung Cancer Adenocarcinoma Transcript 1 (MALAT1), an lncRNA firstly identified in non-small-cell lung cancer, is overexpressed in cervical cancer. Its upregulation, which is caused by HPV infection, promotes proliferation and metastasis, while decreasing the apoptosis of cervical cancer cells [134,137]. MALAT1 can act as a sponge for numerous miRNAs. For example, Han et al. (2019) suggested that, through miR-202–3p, it leads to the activation of the AKT/mTOR signaling pathway [133]. Moreover, MALAT1 promotes cervical cancer growth by inhibiting miR-124, and it was associated with radioresistance in a mechanism linked to the decreased expression of the tumor suppressor miR-145 [135,136].

IDH1-AS1—LncRNA isocitrate dehydrogenase 1 (IDH1) antisense RNA1 (AS1) is known for its action on the inhibition of cell proliferation and on the increase in IDH1 enzymatic activity, thereby increasing the levels of α-ketoglutarate. Upon c-Myc activation, which can be achieved by HPV infection, IDH1-AS1 transcription is inhibited, thus causing the activation of HIF1α and the Warburg phenotype, suggesting that lncRNA IDH1-AS1 may regulate the Warburg effect [138].

LNMICC—LncRNA associated with Lymph Node Metastasis in Cervical Cancer (LNMICC) is associated with cervical cancer metastasis, lymph angiogenesis and poor patient prognosis by acting on fatty acid metabolism [139].

TUG1—The list of relevant cervical cancer-related lncRNAs includes Taurine-Upregulated Gene 1 (TUG1) [112,140]. This lncRNA is reported to regulate mitochondrial bioenergetics by interacting with PGC-1α. This interaction was shown to increase mitochondrial content, mitochondrial respiration, and the ATP levels [141,142].

UCA1—The lncRNA Urothelial Cancer Associated 1 (UCA1), increases cell proliferation and invasion by sponging miR-299–3p [148], miR-145 [146] and by regulating Kinesin Family Member 20A (KIF20A) expression through miR-204 [116] in cervical cancer. UCA1 lncRNA upregulation can also promote cell proliferation and EMT by downregulating miR-155 [147], or by enhancing β-catenin and T-Cell Factor 4 (TCF-4) activity [145]. Interestingly, UCA1 controls glycolysis by modulating the miR-493–5p/ Hexokinase 2 (HK2) axis, highlighting a potential role of this lncRNA in tumor metabolism of cervical cancer [143,144], while it was proposed in the modulation of mitochondrial function in bladder cancer via the miR-195/ARL2 signaling pathway [163].

MEG3—The lncRNA Maternally Expressed Gene 3 (MEG3) levels are significantly reduced in cervical cancer patient serum compared to healthy controls [149]. Its downregulation has been associated specifically to HPV-positive cervical cancer tissues as compared to adjacent normal tissues [150]. The onco-suppressive role of MEG3 was suggested by its overexpression, reducing the level of miR-21-5p expression, in turn causing the inhibition of proliferation and increased apoptosis in cervical cancer cells [150]. Indeed, MEG3 is one of those lncRNAs that enhance mitochondrial apoptosis. In renal cell carcinoma, its high levels may reduce the expression of Bcl-2 and increase the amount of the cleaved caspase-9, and can promote the cytochrome c release from mitochondria [128], a mechanism which activates apoptosis.

WT1-AS—The lncRNA Wilms Tumor 1 Homolog Antisense RNA (WT1-AS) is another tumor suppressor in cervical cancer. High levels of WT1-AS promote apoptosis and suppress proliferation, migration and invasion through the modulation of miR-205 [153]. This lncRNA also modulates p53 expression through the binding of miR-330–5p [151]. It was shown also to limit cervical cancer progression by modulating Forkhead Box N2 (FOXN2) through miR-203a-5p or controlling Phosphoinositide-3-Kinase Adaptor Protein 1 (PIK3AP1) [152,154].

GAS5—Growth-arrest-specific 5 (GAS5) was described as a mitochondrially localized lncRNA tumor suppressor acting in maintaining cellular energy homeostasis and modulating the TCA cycle in breast cancers [164]. Low expression of this lncRNA was reported in cervical cancers, in agreement with its tumor suppressor and apoptosis promoting roles. In cervical cancer cells GAS5 was shown to inhibit the malignancy, proliferation, migration, and invasion, while its low levels favor chemoresistance acting through miR-21- and Akt-mediated mechanisms [155].

SNHG12—Small nucleolar RNA host gene 12 (SNHG12) has been reported to be dysregulated in some types of cancers, including cervical cancer, where it acts as an oncogene [156]. Evidence in cervical squamous cell carcinoma showed that HPV16 E6 and E7 oncoproteins positively regulate the expression level of SNHG12 by modulating the transcription factor c-Myc, thus potentially affecting cell metabolism [156]. Moreover, this lncRNA promotes the EMT, whereas its knockdown in cervical cancer cells decreases proliferation, migration, and invasion and induces apoptosis [156].

### 4.2. MiRNAs in Cervical Cancers

miRNAs regulate essential cellular processes, such as cell proliferation and metabolism under physiological conditions. However, they have been shown to be dysregulated in many cancers [165,166]. During HPV infection different miRNAs are generated, affecting HPV DNA incorporation in cell patient genome and tumor development [111]. Increasing evidence is showing that different miRNA alterations in cervical cancers are under the control of the HPV oncoproteins E5, E6 and E7 [111]. In cervical cancer patients miRNAs act either as oncogenes or as tumor suppressors [112].

#### MiRNAs Modulate Glycolytic and Mitochondrial Metabolism in Cervical Cancers

The roles of different miRNAs that are involved in the development of cervical cancer through the control of mitochondrial and glycolytic pathways are summarized in Table 3.

MiR-16-5p—In cervical cancer, miR-16-5p targets pyruvate dehydrogenase kinase PDK4 to reduce glycolytic metabolic activity and chemical resistance, resulting as a tumor inhibitor [167]. Accordingly, the inhibition of miR-16-5p has been shown to lead to an increased expression of PDK4 and enhanced chemoresistance in cervical cancer [167].

MiR-21—This miRNA has been reported in the context of cervical cancer patient liquid biopsies [112]. MiR-21 upregulation, which occurs in HPV-positive cervical cancer cells, downregulates Reversion-inducing Cysteine-rich protein with Kazal motifs (RECK), increasing cell proliferation, migration and survival [168]. It was shown also to modulate proliferation, migration and apoptosis of cervical cancer cells by interacting with Neurotrophin-3 (NTF3) [169] and its high circulating levels were associated with lymph node metastasis in patients, in line with the activation of EMT process [170].

Moreover, miR-21 overexpression contributes to cell proliferation by inhibiting PTEN [171], which is a tumor suppressor, thus resulting in metabolic reprogramming of cervical cancer cells. In line with this role, Song et al. reported that, in radioresistant cervical tissues, miR-21 overexpression is driven by HIF1α overexpression, where this miRNA downregulates PTEN and increases p-AKT, thus preserving high HIF1α levels [172]. Interestingly in a hypertensive rat model, miR-21 directly targeted the mtDNA-encoded cytochrome b (mt-Cytb) to positively modulate mt-Cytb translation in mitochondria [188].

MiR-21-5p—The oncogenic roles of this miRNA in promoting tumor growth, invasion, and metastasis in cervical cancer were associated to repression of von Hippel–Lindau (VHL) tumor suppressor associated with the HIF-1α stress response pathway [173,174]. Although its role in the modulation of cervical cancer metabolism has not been clarified, miR-21-5p expression was reported to be relevant to mitochondrial and lipid metabolism in a cardiac myoblast model [189].

MiR-34a—This is one of the miRNAs that are under p53 induction. P53 and miR-34a upregulation reduces the expression levels of HK2, pyruvate kinase and HIF1α, thus inhibiting the Warburg effect. Importantly, a negative association between NEAT1 and miR-34a was verified in cervical cancer tissues. Overexpression of miR-34a suppressed the cellular glycolysis rate and sensitized 5-fluorouracil-resistant cells through directly targeting the 3′-untranslated region (UTR) of LDHA, a key glycolytic enzyme [176]. The LDHA targeting by miR-34a was previously found in SiHa and HeLa cell models in which the knockdown of oncoprotein E6 caused an upregulation of miR-34a and a decrease in the Warburg effect [175].

MiR-96—miR-96 has been described as associated with the progression of cervical cancer and its detection was achieved both in cancer cell and patient tissues [112]. Its overexpression enhanced the proliferative, migratory and invasive phenotypes of cervical cancer cells. Moreover, miR-96 upregulation promoted the activation of the AKT/mTOR signaling pathway [177], promoting glucose uptake and utilization. Another target of miR-96 is caveolin-1 (CAV-1), through the decrease level of which, this miRNA can control cell proliferation, migration and invasion [177].

MiR-124-5p–OIP5-AS1 is a hypoxia-responsive lncRNA which was found to be overexpressed in cervical cancer patients. In cervical cancer cells, OIP5-AS1 promotes isocitrate dehydrogenase IDH2 expression through the inhibition of miR-124-5p. In turn, high levels of IDH2 cause a Warburg phenotype in cervical cells under hypoxic condition through HIF1α stabilization and expression [178].

MiR-143—This miRNA was associated with the modulation of Warburg phenotype and HK2 expression level in different tumors [190]. However, in a cervical cancer HeLa cell model, the proliferation rate decreased, while the apoptosis increased in miR-143-expressing groups [179]. The transient upregulation of miR-143 decreased the levels of HIF1α in HeLa cells [179]. Accordingly, miRNA-143 expression negatively correlated with the levels of GOLgi Membrane protein 1 (GOLM1) in cervical cancer tissues, inhibiting cell invasion and migration [180].

MiR-182—This miRNA was shown to increase glucose utilization in muscle by targeting the transcriptional factor Forkhead box O1 (FoxO1) and PDK4, which controls fuel selection via the pyruvate dehydrogenase complex [191]. In cervical cancer cells, the expression of high-risk HPV E7 protein increased the miR-182 expression through the TGF-β/Smad4 signaling pathway, while low-risk HPV E7 did not affect the expression of TGF-β and miR-182. MiR-182 therefore provides a promising prognostic and therapeutic target for the disease [181], as seen for other miRNAs that are selectively expressed upon high-risk HPV infection [111].

MiR-214-5p—Bioinformatics studies revealed the association between lncRNA-TDRG1 and miR-214-5p, and the latter with the transmembrane protein Semaphorin 4C (SEMA4C), as confirmed by a dual-luciferase reporter assay. The study demonstrated that lncRNA-TDRG1 regulated miR-214-5p, which binds to SEMA4C, thus promoting hypoxia-induced glycolysis and cervical cancer growth [182]. In line with this evidence, SEMA4C has been associated with metastatic capacity of tumor cells and poor prognosis in patients [192,193,194].

MiR-214—The upregulation of miR-214, another tumor suppressor in cervical cancer, causes cell death and sensitizes to cisplatin treatment by decreasing Bcl-2-like 2 (Bcl2l2) levels, and increasing those of Bax, caspase-3, caspase-8 and caspase-9 [185]. MiR-214 also represses proliferation, migration and invasion of cervical cancer by negatively regulating ADP Ribosylation factor-Like 2 (ARL2) [183], Mitogen-activated protein Kinase Kinase 3 (MKK3) [184] and Enhancer of Zeste Homolog 2 (EZH2), which serves an important role in regulating cell proliferation [187]. Finally, miR-214 can suppress drug-resistant phenotypes by targeting Forkhead Box M1 (FOXM1) [186].

## 5. Therapy

Current therapies for cervical cancer include surgery, radiotherapy, chemotherapy, or their combinations to achieve improved treatment outcomes, depending on tumor type and stage [195,196]. Indeed, surgery is the primary treatment option for patients with early-stage tumors, concurrent chemoradiotherapy (CRT) is preferred for locally advanced cervical cancer, whereas in case of metastatic, persistent or recurrent cervical cancer, the treatment strategy is decided based on several clinical factors [2,196].

Despite initial therapeutic responses, treatment efficacy is often limited by the marked heterogeneity of cervical tumors, which contributes to radioresistance and chemoresistance [197]. As a consequence, outcomes for patients with advanced or recurrent cervical cancer remain poor, with one-year survival rates below 20% [91].

For these reasons, in addition to conventional treatments, over the past few years, immunotherapy has become an established therapeutic option, particularly for recurrent cervical cancer, and several alternative strategies have also been developed and are currently under investigation [2,195].

### 5.1. Chemotherapy in Cervical Cancers

Chemotherapy is a component of standard management of advanced and recurrent cervical cancer and is used in different clinical settings as a primary treatment for locally advanced disease, as adjuvant therapy following surgery or in combination with radiotherapy [12].

First-line chemotherapy relies on a relatively limited number of agents, with cisplatin being the most commonly used drug. As platinum-based compound, cisplatin triggers apoptosis in cancer cells. Several studies have demonstrated that combining cisplatin with other chemotherapeutic agents enhances antitumor efficacy, helping to overcome resistance and reducing treatment-related toxicity [12,198].

Other chemotherapy drugs, frequently used in combination with cisplatin, are the platinum-based paclitaxel and carboplatin, or agents such as 5-fluorouracil, bleomycin and topotecan. These molecules mainly target pathways causing cell cycle arrest, microtubule network reorganization or inhibition of DNA replication, thereby inducing cell death [12,91].

### 5.2. Immunotherapy Strategies

Thanks to the increased availability of specific tumor biomarkers, immunotherapy has demonstrated the potential to selectively target HPV-associated malignant cells and has exhibited improved outcomes for patients. These approaches, which have the goal to stimulate the body’s immune system, include checkpoint inhibitors, antibody–drug conjugates, therapeutic vaccines, and adoptive T-cell therapies [199]. The benefit of immunotherapy is supported by the results of recent clinical trials, which have reported improved survival rate in patients [2,195,196].

Immune checkpoints represent regulatory pathways exploited by tumor cells to evade immune surveillance. Immune checkpoint inhibitors counteract this process by enhancing T-cell cytotoxicity through a blockade of inhibitory receptors [195]. Common therapies include inhibitors of programmed cell death (PD-1) and its ligand (PD-L1), cytotoxic T-lymphocyte-associated antigen 4 (CTLA-4) and lymphocyte activation gene-3 (LAG-3) [199].

Therapeutic vaccine strategies have been developed to generate T-cell-mediated immunity by specifically targeting the consistently expressed viral oncoproteins, mainly E6 and E7, and numerous trials have shown promising results [199,200].

Furthermore, several Adoptive Cell Transfer (ACT) strategies, such as Tumor Infiltrating Lymphocytes (TILs), Chimeric Antigen Receptor T cells (CAR-T cells) and T Cell Receptor-modified T cell (TCR-T) therapies have also drawn intensive attention [195,199]. For example, clinical trials of TCR-T therapy targeting the E6 or E7 subunits of HPV-16 have shown potential [201,202].

Finally, Antibody Drug Conjugate (ADC) therapy is based on the strategy of linking antibodies to chemotherapeutic agents to increase efficacy and to limit off-target toxicity [195,203]. For cervical cancer, most ADC used are antibodies targeting Tissue Factor (TF) or Human Epidermal Growth Factor Receptor 2 (HER2), combined with strong cytotoxic drugs [203].

### 5.3. Targeted Therapies

Targeted therapies focus on damaging specific molecules and inhibit key signal transduction pathways relevant to cervical cancer progression [195,204].

#### 5.3.1. Angiogenesis Inhibitors

The angiogenesis pathway is a crucial tumorigenic mechanism. The efficacy of the VEGF inhibitor bevacizumab has been demonstrated in various clinical trials, and the combination of this molecule with chemotherapy or radiotherapy has improved treatment outcomes in patients with metastatic or recurrent cervical cancer [205,206]. Other angiogenesis suppressors include tyrosine kinase inhibitors, such as cediranib, apatinib or nintedanib, which have shown promising clinical effectiveness [207,208,209,210].

#### 5.3.2. PARP Inhibitors

Poly(ADP-ribose) polymerases (PARPs), crucial enzymes involved in DNA repair and genome stability, are highly expressed in cervical cancer cells, and their inhibitors, which induce DNA damage and apoptosis, are currently under clinical investigation with encouraging results [211,212].

#### 5.3.3. Metabolic Inhibitors

Several drugs have been studied to target the metabolic reprogramming, in particular glucose and lipid metabolism, and the hypoxia signaling [213,214,215,216]. For example, it has been shown that two inhibitors of FASN, a key enzyme of fatty acid synthesis highly expressed in cervical cancer patients, reduced lymph node metastasis [217]. In vitro studies of cervical cancer cells have shown the potential anticancer activity of drugs that inhibit glucose metabolism leading to apoptosis, either by suppressing the activity of the AKT/mTOR and HIF1α signaling pathways [218] or by the downregulation of key glycolytic enzymes [219]. Moreover, Vosaroxin, a quinolone-derivative anticancer agent, causes mitochondrial dysfunctions and apoptosis in HeLa cells by blocking HIF1 synthesis and complex formation [220]. Interestingly, the efficacy of conventional therapies may be improved through their combinatorial use with these molecules, as demonstrated using glucose uptake blockers, which increase the efficacy of HER inhibitors, overcoming the HIF1α-mediated resistance to HER-targeted therapies [221].

In addition, in vitro studies have explored the antitumor efficacy of natural products [200,214]. Many compounds, including curcumin, resveratrol, berberine, withaferin A, and fig latex, have been shown to suppress HPV16/18 E6 and E7 expression, restoring p53 and pRb signaling, thereby inducing cervical tumor growth arrest and apoptosis [222,223,224,225,226]. Interestingly both curcumin and resveratrol treatments in HeLa cells also inhibited glycolysis, inducing intracellular glucose and lactate reduction, pyruvate augment and cell growth inhibition [227]. Finally, in vitro experiments showed that mycoepoxydiene, a marine fungus product, inhibited cervical cancer cell growth and downregulated the activity of enzymes LDHA and G6PD, which are involved in glycolysis and the pentose phosphate pathway [228].

### 5.4. Genetic Approaches

Genetic approaches represent an innovative strategy and are mainly aimed at targeting the viral sequences of E6 and E7 [195,229]. They include gene editing technologies, such as CRISPR/Cas9, transcription activator-like effector nuclease (TALEN) and zinc-finger nuclease (ZFN) tools, and the RNA interference (RNAi) strategy, which is based on small interfering RNAs (siRNAs) and microRNAs (miRNAs) [111,195,229]. CRISPR/Cas9 technology and siRNA efficacy have been extensively proved using in vitro and in vivo preclinical models [229,230,231,232,233]; moreover, some clinical trials of the TALEN and CRISPR/Cas9 platforms are ongoing to assess safety and efficacy (i.e., NCT03226470 and NCT03057912). Although these approaches have demonstrated potential in suppressing malignant traits [230,231,232], their therapeutic potential remains limited by delivery, immunogenicity, safety and off-target issues [200,229]. MiRNAs represent a potential therapeutic tool, but the use of either miRNA mimics or miRNA inhibitors is hampered by several limitations [111,229]. On the one hand, for a correct delivery, miRNAs need to reach cancer cells crossing the tumor micro-environment, cross the membranes, and escape endosomes, lysosomes and ribonuclease system in order to be active in the cytoplasm. To overcome delivery issues, structural modifications, different nano-vehicle-based systems and viral vector systems are under investigation. On the other hand, the large number of miRNA targets in the human genome may generate undesirable effects, making their clinical application challenging [111]. Similarly, lncRNA-based therapies present some challenges, including off-target effects (epigenetic regulation, post-transcriptional regulation, and interactions with mRNAs and miRNAs), technical delivery issues, stability, immunogenicity, safety and efficacy [234]. For all these reasons, the therapeutic use of lncRNAs and miRNAs remains limited [111,229,234].

## 6. Conclusions

We here recapitulate that cervical cancer is one of the highly occurring cancers in females and presents high incidence and mortality. Persistent infection of HPV is detected in more than 90% of all cervical cancers and is recognized as the primary cause of the disease. Some of the HPV oncogenic proteins, such as E1, E2, E5, E6, and E7, are responsible for the modulation of key factors in the host cell, in turn causing cervical cancerogenesis and metabolic reprogramming. These include p53, c-Myc, HIF1α and several non-coding RNAs, causing cell metabolic rearrangements that are detailed in this review. Furthermore, we give an update of the current therapies that are applied to patients according to the specific stage of cervical cancer, as well as an overview on experimental trials that might, in the future, provide new promising therapies.

## Figures and Tables

**Table 1:** HPV oncoproteins, their specific targets and effects in cervical cancer. Green and red arrows indicate upregulation or downregulation, respectively.

HPV Oncoproteins	Specific Targets	Downstream Effects
**E1**	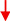 SOD1, SOD2 and GSH	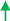 ROS, mitochondrial dysfunction
**E2**	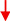 SOD1, SOD2 and GSH Complex III, ATP synthase binding	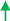 ROS, altered mitochondrial cristae, mitochondrial dysfunction
**E6**	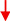 p53 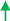 c-Myc	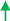 GLUT1/3/4, HK1, HK2, LDHA, MCT1, PDK2 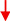 GLS2, CPT1, AIF, ferredoxin reductase
**E7**	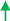 HIF1α 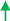 RRM2, PKM2 acetylation ATP synthase binding	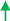 glycolytic flux in hypoxia 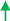 angiogenesis 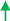 spare mitochondrial respiratory capacity

**Table 2:** **Oncogenic**  **and tumor suppressor lncRNAs and their involvement in cervical cancer.** AKT, Protein Kinase B; ATP, Adenosine Triphosphate; Bcl-2, B-cell Lymphoma 2; EMT, Epithelial–Mesenchymal Transition; FOXN2, Forkhead Box N2; HIF1α, Hypoxia-Inducible Factor 1 Alpha; HK2, Hexokinase 2; IDH1, Isocitrate Dehydrogenase 1; KIF20A, Kinesin Family Member 20A; mTOR, Mechanistic Target of Rapamycin; OXPHOS, Oxidative Phosphorylation; p53, Tumor Protein p53; PGC-1α, Peroxisome Proliferator-Activated Receptor Gamma Coactivator 1 Alpha; PI3K, Phosphoinositide 3-Kinase; PIK3AP1, Phosphoinositide-3-Kinase Adaptor Protein 1; PTEN, Phosphatase and Tensin Homolog; PUMA, p53 Upregulated Modulator of Apoptosis; SIRT1, Sirtuin 1; TCF-4, Transcription Factor 4; Wnt, Wingless-Related Integration Site. In green and red tumor suppressors and oncogenes, respectively.

LncRNA	Oncogene/Tumor Suppressor	Mechanism of Action	Ref.
**ANRIL**	**Oncogene**	- A ctivation of the PI3K/AKT pathway (increased glycolysis, proliferation, metastasis and poor prognosis).	[115]
**CRNDE**	** **Oncogene** **	- Suppression of PUMA signaling and activation of the Wnt/β-catenin pathway (increased proliferation, migration, glycolytic reprogramming and decreased apoptosis).	[118,119,120,121]
**H19**	** **Oncogene** **	- Upregulation of SIRT1 (increased tumor growth and apoptosis inhibition).	[122,123]
**HOTAIR**	** **Oncogene** **	- Activation of PTEN/PI3K/AKT pathway (increased glycolysis, proliferation, metastasis and poor prognosis). - Modulation of Bcl-2 (suppressed apoptosis).	[124,125,126,127,128,129,130,131]
**LincRNA-p21**	** **Oncogene** **	- Interaction with HIF1α (regulation of angiogenesis, mitochondrial metabolism, tumor invasion, and hypoxia-induced glycolysis).	[132]
**MALAT1**	** **Oncogene** **	- Activation of the AKT/mTOR pathway (increased glycolysis, cell proliferation and metastasis with apoptosis inhibition).	[133,134,135,136,137]
**IDH1-AS1**	** **Tumor suppressor** **	- Enhancement of IDH1 activity and α-ketoglutarate production (cell proliferation inhibition and metabolic homeostasis regulation).	[138]
**LNMICC**	** **Oncogene** **	- Reprogramming of fatty acid metabolism (promotion of metastasis and poor prognosis).	[139]
**TUG1**	** **Oncogene** **	- Interaction with PGC-1α (increased mitochondrial content and ATP production through OXPHOS).	[140,141,142]
**UCA1**	** **Oncogene** **	- Regulation of KIF20A and HK2 expression, and enhancement of β-catenin/TCF-4 signaling (increased cell proliferation, invasion, EMT and glycolysis).	[116,143,144,145,146,147,148]
**MEG3**	** **Tumor suppressor** **	- Downregulation of Bcl-2 and promotion of cytochrome c release (increased apoptosis). - Proliferation inhibition.	[128,149,150]
**WT1-AS**	** **Tumor suppressor** **	- p53, FOXN2 and PIK3AP1 regulation (apoptosis promotion and suppression of cell proliferation and migration).	[151,152,153,154]
**GAS5**	** **Tumor suppressor** **	- TCA cycle modulation. - Promotion of apoptosis. - In hibition of proliferation, migration, and invasion.	[155]
**SNHG12**	** **Oncogene** **	- Regulated by c-Myc. - EMT promotion.	[156]

**Table 3:** **Oncogenic and tumor suppressor miRNAs and their involvement in cervical cancer.** AKT, Protein Kinase B; ARL2, ADP Ribosylation Factor-Like GTPase 2; Bax, Bcl-2-Associated X Protein; Bcl2l2, B-cell Lymphoma 2-Like 2; CAV-1, Caveolin-1; EZH2, Enhancer of Zeste Homolog 2; FOXM1, Forkhead Box M1; GOLM1, Golgi Membrane Protein 1; HIF1α, Hypoxia-Inducible Factor 1 Alpha; HK2, Hexokinase 2; IDH2, Isocitrate Dehydrogenase 2; LDHA, Lactate Dehydrogenase A; MKK3, Mitogen-Activated Protein Kinase Kinase 3; mTOR, Mechanistic Target of Rapamycin; NTF-3, Neurotrophin-3; p-AKT, Phosphorylated Protein Kinase B; PDK4, Pyruvate Dehydrogenase Kinase Isozyme 4; PTEN, Phosphatase and Tensin Homolog; RECK, Reversion-Inducing Cysteine-Rich Protein with Kazal Motifs; SEMA4C, Semaphorin 4C. In green and red tumor suppressors and oncogenes, respectively.

MiRNA	Oncogene/Tumor Suppressor	Mechanism of Action	Ref.
**miR-16-5p**	** **Tumor suppressor** **	- Targeting of PDK4 (glycolysis inhibition and reduced chemoresistance).	[167]
**miR-21**	** **Oncogene** **	- Downregulation of RECK (increased cell proliferation, metastasis and survival). - Interaction with NTF3 (modulation of proliferation, migration and apoptosis). - PTEN inhibition and upregulation of p-AKT and HIF1α (increased cell proliferation and metabolic reprogramming promotion).	[112,168,169,170,171,172]
**miR-21-5p**	** **Oncogene** **	- Repression of VHL tumor suppressor (increased growth, invasion and metastasis).	[173,174]
**miR-34a**	** **Tumor suppressor** **	- HK2, pyruvate kinase, HIF1α and LDHA inhibition (decreased Warburg effect).	[175,176]
**miR-96**	** **Oncogene** **	- Activation of the AKT/mTOR signaling pathway (promoted glucose uptake and utilization). - Inhibition of CAV-1 (regulation of cell proliferation and invasion).	[112,177]
**miR-124-5p**	** **Tumor suppressor** **	- Inhibition of IDH2 (decreased glycolysis).	[178]
**miR-143**	** **Tumor suppressor** **	- Downregulation of HIF1α (metabolic reprogramming). - Downregulation of GOLM1 (inhibition of cell invasion and migration).	[179,180]
**miR-182**	** **Oncogene** **	- Increased expression through the TGF-β/Smad4 signaling pathway.	[181]
**miR-214-5p**	** **Tumor suppressor** **	- Binding with SEMA4C (regulation of glycolysis and cell growth).	[182]
**miR-214**	** **Tumor suppressor** **	- Downregulation of Bcl2l2 and upregulation of Bax, caspase-3, caspase-8 and caspase-9 (increased cell death). - Downregulation of ARL2, MKK3 and EZH2 (decreased proliferation, migration and invasion). - Targeting of FOXM1 (suppression of drug resistant-phenotype).	[183,184,185,186,187]

## Data Availability

No new data were created or analyzed in this study.
